# Apicoectomy of Perforated Root Canal Using Bioceramic Cement and Photodynamic Therapy

**DOI:** 10.1155/2020/6677588

**Published:** 2020-12-09

**Authors:** Amjad Abu Hasna, Daiane Pereira Santos, Tania Regina Gavlik de Oliveira, Alana Barbosa Alves Pinto, César Rogerio Pucci, José Luiz Lage-Marques

**Affiliations:** ^1^Department of Restorative Dentistry, Endodontics Division, Institute of Science and Technology, Sa˜o Paulo State University—UNESP, Sa˜o José Dos Campos, SP, Brazil; ^2^Faculty of Dentistry, São Leopolodo Mandic, São Paulo, SP, Brazil; ^3^Department of Dental Materials and Prosthodontics, Institute of Science and Technology, Sa˜o Paulo State University—UNESP, Sa˜o José Dos Campos, SP, Brazil; ^4^Department of Restorative Dentistry, Institute of Science and Technology, Sa˜o Paulo State University—UNESP, Sa˜o José Dos Campos, SP, Brazil; ^5^Department of Restorative Dentistry, School of Dentistry, University of São Paulo, São Paulo, SP, Brazil

## Abstract

Root perforation is a common endodontic accident. Its management depends mainly on root canal disinfection and sealing the perforation area by preventing any communication with the periodontium to prevent recontamination. A patient was referred to treat root perforation due to a previous treatment of tooth #22. The diagnosis was symptomatic periapical periodontitis, and the treatment plan was to retreat the root canal of #22 and make a surgical intervention (apicoectomy) associated with antimicrobial photodynamic therapy as a complementary technique. Five mineral oxides (5MO) cement was used as a root-end filling material. The procedures were performed in two sessions and controlled in two visits (after 30 days and 12 months). A bone neoformation was observed at the periapical area of tooth #22. 5MO bioceramic cement was effective in inducing the repair of the periapical lesion and had the ability to seal the exposed periapical area of the tooth. Its success depended mainly on root canal and surgical site disinfection.

## 1. Introduction

The symptomatic periapical periodontitis is an endodontic disease of the necrosed pulp and its management depends mainly on root canal disinfection [1] in which the manual/automated instruments remove the microorganisms mechanically and the auxiliary chemical substances act chemically [2–4]. However, in some cases, additional complementary techniques are indicated to provide more favorable decontamination using photodynamic therapy (PDT) [5, 6] and passive ultrasonic irrigation (PUI) [7, 8]. As well, the endodontic irrigants play a principal role in reducing torsional and fatigue resistance during instrumentation [9].

During the endodontic treatment [10], some complications like root chamber or root canal perforation may occur because of operative procedural accidents [11] which may be related to lack of experience of the professional [12] or pathological factors [13]. Endodontic perforation results in communicating the root canal system with the periodontium that may lead to tooth loss unless good management is carried out [14].

Endodontic (paraendodontic) surgery was started in the last century as the last alternative of endodontic intervention [15]. It results in satisfactory treatment outcomes and lesion regression [16] without clinical signs and symptoms of inflammation [17].

The bioceramic cements played a major effect on the success of endodontic surgery, since the introduction of mineral trioxide aggregate (MTA) [14, 18–20], Biodentine [21], and lastly the five mineral oxides (5MO) [16, 22].

The photodynamic therapy is a complementary technique, which aids in additional disinfection using a light source like laser or light-emitting diode (LED) acting over a photosensitizer and thus liberating reactive oxygen specimens that disinfect some facultative microorganisms [23] and resulting in more accelerated tissue repair [5, 24, 25].

The aim of this case report was to evaluate the effect of 5MO bioceramic cement on periapical lesion repair and its ability to seal the exposed periapical area of the tooth.

## 2. Case Report

### 2.1. Case Presentation and Patient Information

A Brazilian white 31-year-old male was indicated to treat the left upper lateral incisor #22. The patient stated a “bad experience” with the root canal treatment and a “continuous discomfort”. The patient's clinical history did not present relevant findings.

The clinical examination revealed a positive response to percussion and digital palpation in the periapical region of #22 with no fistula. The intraoral examination did not show any caries or color alteration related to the respected tooth. The depth of its gingival pocket varied between 1 and 3 mm with various exploring locations and grade I mobility. Teeth 21–23 were tested by the pulp vitality test (the cold test) performed by refrigerant gas (Endo Ice, Maquira Dental products industry LTDA, Brazil) and relative isolation using cotton rolls and a dental saliva ejector. Teeth 21 and 23 presented positive responses with characteristics of healthy pulp tissue. However, tooth 22 had a negative response [26].

**Figure 1:**
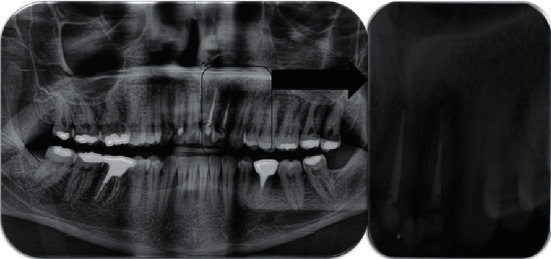
Initial panoramic and periapical radiography of the periapical lesion of tooth #22.

**Figure 2:**
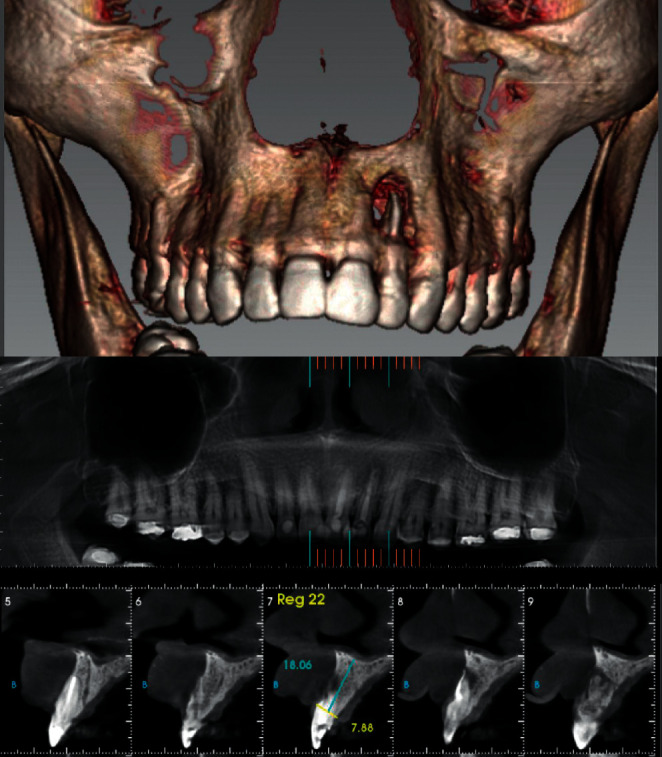
Initial CBCT images of the periapical lesion of tooth #22.

Panoramic and periapical radiographic examination revealed a radiolucent circumscribed lesion around the periapical region of tooth #22 presenting features of periapical periodontitis. As well, unsatisfactory endodontic treatment of the same tooth was founded with signs of root perforation due to a previous treatment (Figure 1). The cone beam computed tomography (CBCT) scan was indicated to obtain an accurate diagnosis of the lesion and its relationship with the adjacent teeth and to confirm the presence of the root canal perforation (Figure 2).

The final diagnosis was symptomatic periapical periodontitis, and the treatment plan was to retreat the root canal and posteriorly to perform an endodontic surgery (apicoectomy), disinfect the periapical region by antimicrobial photodynamic therapy (aPDT), and seal the perforation area with bioceramic cement to prevent recontamination of the canal.

### 2.2. Therapeutic Interventions

Firstly, the root canal of tooth #22 was retreated to disinfect the contaminated system because of the perforation. The canal was instrumented with the RECIPROC system R40/0.06 file (VDW, Munich, Germany) and irrigated by sodium hypochlorite 2.5% (Biodinâmica, Ibiporã, PR, Brazil) and ethylenediaminetetraacetic acid with detergent (EDTA-T) (Fórmula e Ação, São Paulo, SP, Brazil). The canal was then washed with 10 mL of sterile saline solution to be neutralized of any chemical substance and dried with paper points #40.

The aPDT was then performed by filling the canal with methylene blue 0.005% (Vetec Quimica Fina Ltda, Rio de Janeiro, RJ, Brazil) and maintained in the canal for 5 min as a preirradiation time. The irradiation procedure was performed using a low-power diode laser (MMOptics Ltda, São Carlos, Brazil) and an optical fiber (0.40 mm diameter and 16 ± 0.5 mm active surface length) placed into the canal. The irradiation was performed by a visible red wavelength of 660 nm and an output power of 100 mW/cm^2^ activated for 2 min without interval, using a helical movement from apical to cervical direction. An energy density of approximately 120.0 J/cm^2^ was applied [27].

The root canal was obturated in the same session with gutta-percha and Ah Plus sealer (Dentsply, DeTrey GmbH, Konstanz, Germany). No medication was prescribed before or during the treatment. The patient was advised to take acetaminophen (500 mg, maximum four times a day) in case of pain.

One week later, after tomographic planning, the apicoectomy surgery was performed under local anesthesia using one anesthetic tube (4% articaine with epinephrine 1: 100,000), with intraoral access to the lesion achieved via intrasulcular incision of the buccal region from teeth 11 to 24. After detachment of the flap, the perforation was clear and a minimum osteotomy was performed to obtain a surgery window using a surgical carbide drill no. 06 (Angelus Prima Dental Ltda., Londrina, PR, Brazil) under intense irrigation with sterile saline solution.

**Figure 3:**
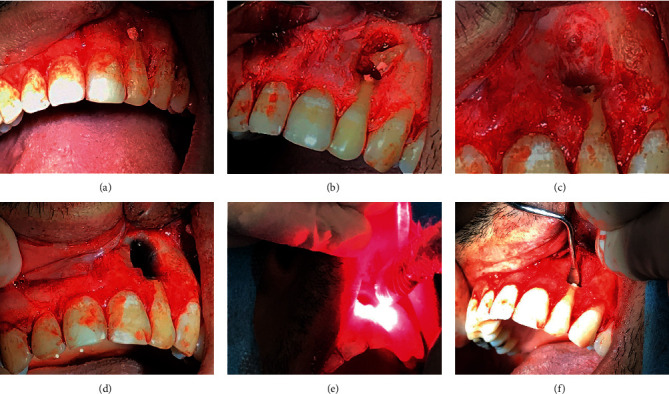
The surgical intervention. (a) Surgical window; (b) apicoectomy; (c) after retroinstrumentation; (d) methylene blue application; (e) laser irradiation; (f) retrofilling with 5MO cement.

**Figure 4:**
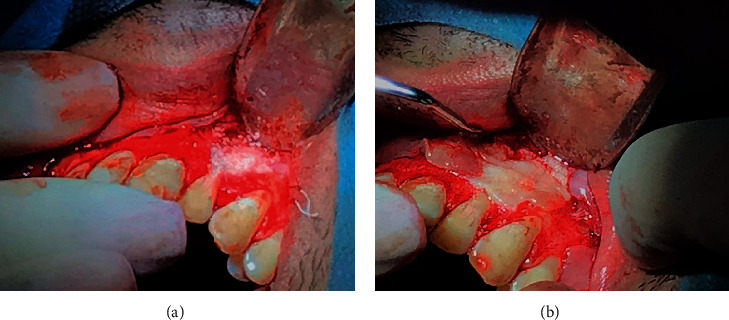
The surgical intervention. (a) Bone graft positioning and (b) membrane placement.

**Figure 5:**
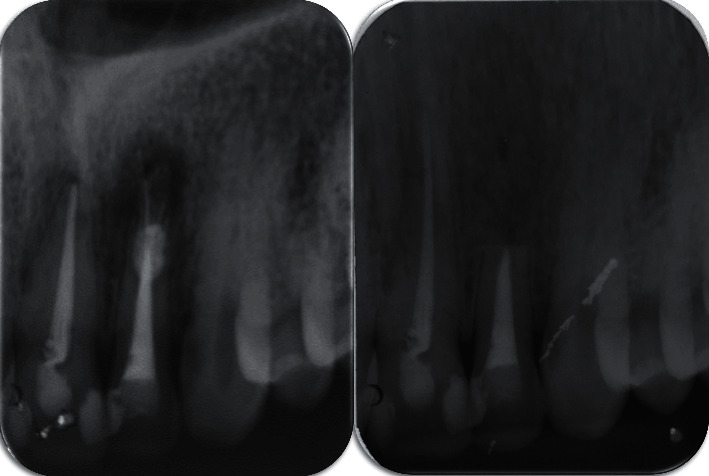
Postoperative periapical radiography immediately after suturing, compared with the initial one.

The apical third of the root was sectioned using Zekrya Surgical Bur (Dentsply, DeTrey GmbH, Konstanz, Germany) under intense irrigation with sterile saline solution, and then, the root canal was retroinstrumented by ultrasonic diamond tip P1 (Helse Ultrasonic, Santa Rosa de Viterbo, SP, Brazil) using CVDentus 100 ultrasound activator (CVDentus, São José dos Campos, Brazil). The surgery site was filled with methylene blue 0.005% and irradiated with a low-power diode laser following the same protocol described above to obtain further decontamination. Then, the root canal was retrofilled with 5MO bioceramic cement (SHAM Dentico, Oman) manipulated following the manufacturer's guidelines (Figure 3). Finally, a bone graft and membrane were placed over the surgery window (Figure 4) to accelerate the periapical lesion bone regeneration [28], and the flap was repositioned, followed by intrasulcular suturing with 3-0 silk thread (Procare Xuyi Webest Medical Products Co, Jiangsu, China). Postoperative periapical radiography was performed immediately after suturing (Figure 5). The suture was removed ten days later, and the patient progressed well postoperatively without intercurrences.

### 2.3. Follow-Up and Outcomes

Ten days later, the patient had no postoperative intercurrences and an intraoral evaluation was performed to investigate any hematoma or edema. The patient related a slight edema in the first two days following the surgery that disappeared posteriorly. No exudate was observed or related.

**Figure 6:**
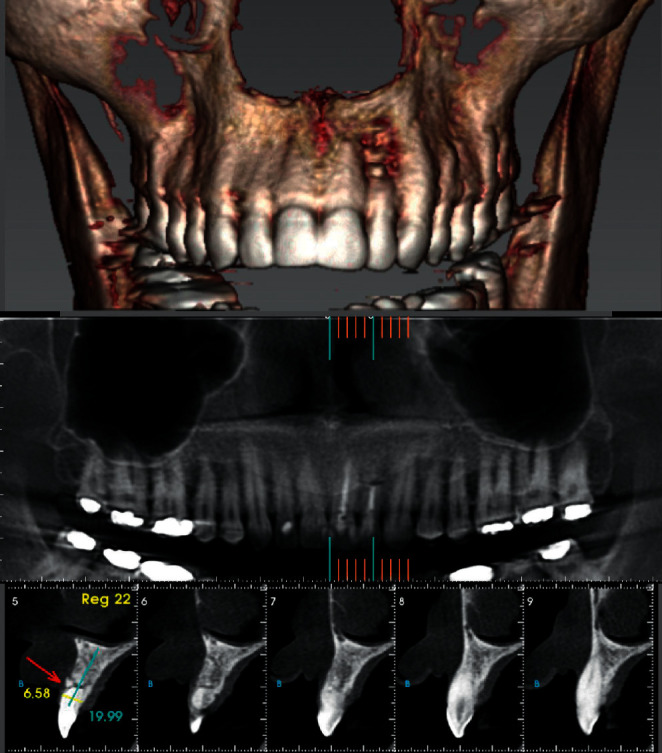
CBCT images of the periapical lesion of tooth #22 after 12 months.

Two follow-up sessions were performed after 30 days and 12 months. In these sessions, clinical intraoral examination was performed in addition to radiographic examination. In the second session (12 months later), the patient progression was evaluated by CBCT images and a bone neoformation was observed at the periapical area of tooth #22 (Figure 6).

## 3. Discussion

The diagnosis of such cases has a direct effect on the treatment plan and outcome. In this case, the CBCT scan was indicated as it provides a three-dimensional mapping of the lesion and its relationship with the adjacent teeth and anatomical structure [29]. The periapical and panoramic radiography is less accurate, and the apicoectomy may not be performed based on their data [30].

The treatment plan was to retreat the infected tooth and associate this retreatment with surgical intervention, as the conventional endodontic retreatment alone is effective in limited cases, and this depends mainly on perforation type, its location, and the professional ability to approach it for repair [31]. However, surgical intervention has a good prognosis when followed by apical sealing with root-end fillings [32].

In this case report, 5MO cement was used as it presented a reparative feature and provided sealing of surgical areas [16]. However, not only 5MO but also many bioceramic cements principally mineral trioxide aggregate (MTA) and Biodentine have the same features [19, 33]. MTA was indicated many years ago as an effective root-end filling material due to its biocompatibility and sealing ability [18], and since then, many bioceramic cements with the same intention presented similar results [22, 34]. As well, modified MTA forms were indicated for high stress-bearing areas and especially for surgical sites [35].

Another point to emphasize is the marginal adaptation of the retrofilling materials. Amalgam as an example was criticized and not indicated due to its expansion over time regardless of its thickness [36]; conversely, MTA has better adaption compared to amalgam and other materials [37]; even more, an improved stability of marginal adaptation of bioceramics over time was proved in a more recent study [38].

In the literature, bioceramic cements have low antimicrobial action; however, this ability to obtain reasonable results in terms of successful management of endodontic complications and accidents is related to the disinfection of the surgical site and root canal in the first place [39]. In this case report, the root canal was instrumented with the RECIPROC system [40] and irrigated with sodium hypochlorite which was proved as an effective antimicrobial agent over resistant microorganisms and its endotoxins [3, 8] and over the matrix metalloproteinases (MMPs) [41] responsible of extracellular matrix degradation and tissue destruction in apical periodontitis lesions [42, 43].

The apical third was retroinstrumented using the ultrasonic tip which is an effective method resulting in additional disinfection of the surgical site [44]. A diamond ultrasonic tip was used as it provokes a minimal number of postoperative cracks when compared to stainless steel tips [45].

Even more, aPDT was used both in root canal retreatment as it was indicated as an effective complementary technique over resistant microorganisms [6] and in the surgical site because it induces reparation of the lesion [5].

Lastly, the endodontic infection has a complex nature, as diverse microorganisms, endotoxins, MMPs, and growth factors are involved [46]. Thus, the combined effect of the disinfection protocols of the endodontic retreatment using an effective instrumentation system, chemical agents, and complementary techniques in addition to the use of a good periapical sealer like bioceramic cements results in infection control of such cases.

## 4. Conclusion

5MO bioceramic cement induces repair of periapical lesion, has the ability to seal the exposed periapical area of the tooth, and has a good marginal adaptation. The success obtained in this case depended mainly on root canal and surgical site disinfection by the photodynamic therapy.

## Data Availability

No data were used to support the findings of this study.
